# Cost-efficiency assessment of Advanced Life Support (ALS) courses based on the comparison of advanced simulators with conventional manikins

**DOI:** 10.1186/1471-227X-7-18

**Published:** 2007-10-22

**Authors:** José Antonio Iglesias-Vázquez, Antonio Rodríguez-Núñez, Mónica Penas-Penas, Luís Sánchez-Santos, Maria Cegarra-García, Maria Victoria Barreiro-Díaz

**Affiliations:** 1 Public Emergency Medical System of Galicia-061 (PEMSG), Servicio Galego de Saúde (SERGAS), Santiago de Compostela, Spain; 2 Pediatric Emergency and Critical Care Division, Department of Pediatrics, Hospital Clínico Universitario de Santiago de Compostela, Servicio Galego de Saúde (SERGAS) and University of Santiago de Compostela, Spain; 3 Arzúa's Primary Care Center, Servicio Galego de Saúde (SERGAS), Arzúa, A Coruña, Spain

## Abstract

**Background:**

Simulation is an essential tool in modern medical education. The object of this study was to assess, in cost-effective measures, the introduction of new generation simulators in an adult life support (ALS) education program.

**Methods:**

Two hundred fifty primary care physicians and nurses were admitted to ten ALS courses (25 students per course). Students were distributed at random in two groups (125 each). Group A candidates were trained and tested with standard ALS manikins and Group B ones with new generation emergency and life support integrated simulator systems.

**Results:**

In group A, 98 (78%) candidates passed the course, compared with 110 (88%) in group B (p < 0.01). The total cost of conventional courses was €7689 per course and the cost of the advanced simulator courses was €29034 per course (p < 0.001). Cost per passed student was €392 in group A and €1320 in group B (p < 0.001).

**Conclusion:**

Although ALS advanced simulator systems may slightly increase the rate of students who pass the course, the cost-effectiveness of ALS courses with standard manikins is clearly superior.

## Background

Cardiopulmonary resuscitation (CPR) training needs specific courses built on experienced instructors, adequate lectures, skill-stations and the simulation of realistic scenarios. The availability of specifically designed manikins and the implementation of learning systems and course regulations, following Resuscitation Council's recommendations, have allowed for the effective training of a huge number of health care professionals [1-8].

Simulation is a general term for an interactive educational strategy that has been shown to be highly useful in the qualification of professionals working in emergency conditions (like flight pilots and physicians) [9-12]. Similarly, new simulation systems, including sophisticated manikins and computers, represent an important step ahead in technology as well as in medical training possibilities.

The theoretical advantages of "simulation system manikins" include: real-time records of the scenario (including responses or treatments applied by the student to the patient) allowing for a more accurate debriefing, more sophisticated manikin features (central and peripheral pulses, real cardiac and respiratory sounds, improved airway simulation etc.) and connection to a personal computer that permits the design and execution of an array of closed and open scenarios according to the trainings needs of courses, instructors and candidates [13-16].

The introduction and development of simulation models in health care professional's training programs should attempt to improve the quality of training and, subsequently, clinical practice. However there is currently little evidence of this. Today simulation systems are very expensive [17] and doubts exist about whether they are worthwhile in cost-effective terms and in terms of the quality of the training process compared to usual ALS teaching materials and courses.

The aim of the present study is to assess the impact of the introduction in ALS courses of last-generation simulation system in terms of cost-effectiveness.

## Methods

The Public Emergency Medical System of Galicia-061 (PEMSG) has a teaching center in charge of practical training of all PEMSG health care staff (emergency medical technicians, nurses and physicians). It also offers courses for other health care professionals (primary care doctors and nurses, pediatricians and other medical specialists) as well as courses for (e.g.) the police or fire-brigade ALS courses are mandatory for PEMSG nurses and physicians and they follow the course regulations (in terms of programs, teaching material and instructors) recommended by the European Resuscitation Council (ERC) and the Spanish Resuscitation Council (SRC).

Two hundred fifty students admitted to ALS courses were divided in two groups: 125 candidates were assigned to 5 courses that followed the "standard rules" and used conventional ALS manikins (ALS Skilltrainer^® ^with Heartsim 200^® ^arrhythmia simulator, designed by the Laerdal Company, Stavanger, Norway) (group A) and the remaining 125 were assigned to 5 courses based on scenarios supported by the brand new SimMan^® ^simulator (software version 2.1), also designed and manufactured by Laerdal Company, Stavanger, Norway (group B). The manikins main characteristics appear in table 1.

**Table 1:** Manikins' characteristics

	**ALS TRAINER**^**®**^	**SimMAN**^**®**^
Intubation	Yes	Yes
Difficult airway	No	Yes
Pneumothorax	No	Yes
ECG Monitoring	Yes	Yes
Venous access	Yes	Yes
Remote control	No	Yes
Pulse check	Carotid	Carotid, femoral, radial
Cardiac rhythms	30	2.500*
Blood pressure*	No	Yes
Both sex genitals*	No	Yes
Parametric monitor*	No	Yes
Case register*	No	Yes

* Features not essential for the ALS course objectives.

Candidates were assigned to groups at random, and the course programs (with the exception of manikins used) were similar in both groups. The duration of the course was 20 hours over four days (5 hours/day) with a ratio of lectures to practical sessions of 1:1 (practical tests not included). The number of trainees per course was 24–26, with 4 instructors (student/instructor ratio of 6:1), one course director and one person in charge of the equipment. The schedule was designed according to teaching programs currently recommended by major CPR associations [18,19]. Nurses and physicians were present and mixed in all courses and requirements to pass the course were the same for all candidates.

All skill stations and test scenarios were designed beforehand using CPR instructor manuals [18,20,21]. They included predefined flow charts, with acceptable and unacceptable responses, specific points to be addressed and the minimum number of responses accepted to pass the practical test. Borderline pass candidates at scenario test were re-tested by another instructor. Cut-off point to pass written test was 85%.

A total of 10 expert instructors participated in the courses. All of them had passed at least one ALS instructor course, had participated in at least in 3 prior ALS courses and had also received specific training on the use of the SimMan^® ^simulator, including its periodic updates during the study period (years 2003 and 2004). The course director has extensive experience at national and international level in ALS, pediatric advanced life support and advanced trauma life support courses.

The cost calculations for the courses were made by the PEMSG accounting staff. They were unaware of the object of this study and have considered all the used material (property of the center, renting, disposable equipment and pharmacy material), educational material, secretarial duties, instructors pay and other additional costs (like transport, classrooms, etc.).

Instructors received 48 euros for each lecture and 30 euros for each skill station. In the structural costs we included the property costs of the educational services of our educational center (personnel, place conditioned for the formative activity, etc.). We also included the common costs derived from the day to day running of the foundation generated by the course (cleaning, communications, supplies, management, etc.). We imputed a proportional form to the weight of the personnel of the educational center inside the Foundation, which represent 2.5%.

For the material property of the center we considered depreciation and maintenance costs. We included in every course the proportional part of costs depending on the number of effective utilization hours of the equipment. For renting we considered the rent cost of three classrooms for 10 practical hours and one theoretical classroom with capacity for 30 pupils equipped with projection screen, table, and auxiliary material. We also included three classrooms necessary for practical stations. Renting cost of manikins was calculated considering cost of product, maintenance and number of uses during the redemption period. Results are expressed in euros.

### Statistical analysis

Results are presented as number and percentage or mean ± standard deviation. Statistical testing were done with Chi-squares test for categorical and Student's t-test for continuous data, as appropriate. A p < 0.05 was considered significant.

## Results

Candidates of both groups were similar in terms of age, sex, previous training and employment status (Table 2).

**Table 2:** Candidates characteristics

	**Group A**	**Group B**	**P**
Students	125	125	
Men/women*	58/67	49/76	n.s.
Physicians*	61	68	n.s.
Nurses*	64	57	n.s.
Age (years)#	41 ± 6,3	44 ± 8,1	n.s.
Prior ALS courses*	42	37	n.s.
Courses in last two years*	31	29	n.s.

• * Chi-square test

• # Student's t test

In group A (conventional training) 98 students (78%) passed and 27 (22%) failed the course. In contrast, in group B (new simulator training) 110 students (88%) passed and 15 (12%) failed the course (p = 0.06).

The total cost of course B (€ 29034 per edition) was significantly higher than cost of course A (€ 7689 per edition) (p < 0.001). The items that account for the cost difference between courses were "material property of the center" (22391 vs. 1599 euros, p < 0.001) and "renting" (275 vs 75 euros, p < 0.01) (Table 3).

**Table 3:** Costs of the courses (in euros).

	**Group A**	**Group B**	**p**
Editions	5	5	
Material property of the center	1599	22391	<0.001
Renting	75	275	<0.001
Disposable equipment	80	85	n.s
Pharmacy	12	12	n.s.
Educational material	37	45	n.s.
Accreditation	15	15	n.s.
Instructors payement	1380	1420	n.s
Structural cost	4490	4790	n.s.
**Total cost**	**7689**	**29034**	**<0.001**

When considering cost per passed student, the results were € 1320 in course B and € 392 in course A (p < 0.001).

Also, teaching costs per candidate were higher in course B (€ 1161) than course A (€ 308) (p < 0.001).

## Discussion

The Best Evidence Medical Education (BEME) Collaboration systematic review of high-fidelity simulation studied the characteristics and uses of medical simulations that lead to most effective learning, identifying ten key features of medical use of simulation: providing feedback, repetitive practice, curriculum integration, range of difficulty level, multiple learning strategies, capture clinical variation, controlled environment, individualized learning, defined outcomes and simulator validity [22].

Simulation systems that include advanced manikins and computer represent a qualitative step ahead in teaching technology as well as in training possibilities for emergency healthcare staff.

Although same closed or open scenarios can be used, and the critical points for scenario resolutions use similar standards for the ALS trainer^® ^and SimMan^®^, when compared with standard manikin-based courses, integrated simulators also offer very realistic scenarios and provide the possibility to do in depth debriefing as well as improved feed-back [23-25]. This is important because feed-back is a crucial component of the learning process associated to simulation that can be improved in quality with in depth debriefing of the scenario performed by means of candidates and instructor interaction.

Previous concerns have been expressed about efficiency of simulation systems [26,27]. In this way the use of advanced systems like SimMan^® ^for the purposes of ALS courses may have relevant drawbacks. Simulators are very expensive even for wealthy training centres; then a significant amount a budget must be devoted to the purchase and maintenance of sufficient quantities of manikins. This fact has limited in many cases the teaching possibilities of centers, especially in low income areas or in non-public funded institutions [28-30]. New simulation systems will challenge the budgets of teaching centers and might hinder the global objective of training and retraining all healthcare staff in advanced life support [31-34].

In addition, simulation needs expert instructors with the ability to simultaneously interact with the students and the simulator. To achieve this expertise many work hours are needed and a learning curve must be considered. This fact could limit the access of interested candidates to courses. In addition, some SimMan^® ^features that contribute to its high cost are not essential to fulfill the specific ALS course objectives.

Taking into account the theoretical virtues and drawbacks, is it worthwhile to implement SimMan^® ^or other advanced simulators in ALS courses?.

In this sense, and as far as we know, our study is the first to assess from a practical point of view the comparative cost-effectiveness of an advanced simulator in ALS courses. Our results are quite clear and indicate that, although simulator systems can slightly increase the rate of passed candidates (from 78% to 88%) it is achieved at a high cost (3.77 times when total expenses are considered and 3.35 times if related to the cost per passed student).

Our study has limitations that must be considered. We have not included the costs derived from instructor's specific training to manage simulation systems [31]. This expense is very difficult to estimate accurately and will increase the cost difference in favor of standard manikins. Our results could be influenced by some differences in manikin performance and their capability to practice and measure the depth of chest compressions. In this sense our instructors and candidates did not make objections and we consider that such differences had a minimal influence on results.

Despite the possible disadvantage of simulation in cost-effectiveness for ALS courses, we consider that simulation may have an outstanding role in medical training including emergency, critical care, trauma, surgery and general and nurse procedures. In our opinion, significant effort should be made by manufacturers in order to produce high quality products at a reasonable price that permit wide implementation of medical simulation [35-41].

Finally, our results should not be generalized because they were obtained in a specific environment and in a public medical emergency system and it is possible that a similar study, carried out in institutions with different characteristics, would give different results. We encourage such studies.

## Conclusion

New medical simulation systems are effective training tools for ALS courses but they are not worthwhile, in terms of cost, when compared to ALS courses based on conventional manikins.

## Competing interests

The author(s) declare that they have no competing interests.

## Authors' contributions

JAIV conceived the study, participated in the design of the study and drafted the manuscript. ARN and LSS participated in the design of the study, statistical analysis and reviewed the manuscript. MPP worked with all the cost study and the design of the economical part of the study. MCG and MVBD participated in the design and reviewed the manuscript. All authors read and approved the final manuscript.

## Pre-publication history

The pre-publication history for this paper can be accessed here:


